# Investigation on the performance of dedicated radiotherapy positioning devices for MR scanning for prostate planning

**DOI:** 10.1120/jacmp.v16i2.4848

**Published:** 2015-03-08

**Authors:** Jidi Sun, Jason A. Dowling, Peter Pichler, Joel Parker, Jarad Martin, Peter Stanwell, Jameen Arm, Fred Menk, Peter B. Greer

**Affiliations:** ^1^ School of Mathematical and Physical Sciences, University of Newcastle Newcastle NSW Australia; ^2^ CSIRO Australian E‐Health Research Centre Brisbane QLD Australia; ^3^ Radiation Oncology, Radiation Oncology Department Calvary Mater Newcastle Newcastle NSW Australia; ^4^ School of Health Sciences, School of Health Sciences, University of Newcastle Newcastle NSW Australia; ^5^ Hunter New England Health Calvary Mater Newcastle NSW Australia

**Keywords:** treatment planning, prostate cancer, MR‐based planning, image quality, patient positioning

## Abstract

The purpose of this study was to investigate performance of the couch and coil mounts designed for MR‐simulation prostate scanning using data from ten volunteers. Volunteers were scanned using the standard MR scanning protocol with the MR coil directly strapped on the external body and the volunteer lying on the original scanner table. They also were scanned using a MR‐simulation table top and pelvic coil mounts. MR images from both setups were compared in terms of body contour variation and image quality effects within particular organs of interest. Six‐field conformal plans were generated on the two images with assigned bulk density for dose calculation. With the MR‐simulation devices, the anterior skin deformation was reduced by up to 1.7 cm. The hard tabletop minimizes the posterior body deformation which can be up to 2.3 cm on the standard table, depending on the weight of volunteer. The image signal‐to‐noise ratio reduced by 14% and 25% on large field of view (FOV) and small FOV images, respectively, after using the coil mount; the prostate volume contoured on two images showed difference of $1.05\pm 0.66\;{\text{cm}}^{3}$. The external body deformation caused a mean dose reduction of 0.6±0.3 Gy, while the coverage reduced by 22%±13% and 27%±6% in $V_{98}$ and $V_{100}$, respectively. A dedicated MR simulation setup for prostate radiotherapy is essential to ensure the agreement between planning anatomy and treatment anatomy. The image signal was reduced after applying the coil mount, but no significant effect was found on prostate contouring.

PACS numbers: 87.55.D‐, 87.61.‐c, 87.57.C‐

## I. INTRODUCTION

The objective of prostate radiotherapy is to accurately deliver uniform dose to the prostate while keeping the radiation toxicity in the nearby normal tissue and organs at risk at a minimum level.^(1)^ The localization of organs is critical for accurate target planning and tissue sparing. Modern structural image modalities, such as CT and MR images, are employed for the delineation of the organs of interest. As human bones have a higher atomic number than soft tissue, CT imaging has an advantage in identifying bony structures. However, the contrast between soft tissues is not high on CT. This disadvantage can result in a larger planning target volume margin during the prostate delineation and, in turn, reduces the normal tissue sparing.^2^, ^3^, ^4^ Steenbakkers et al.^(3)^ found that the MRI generated plan was able to maintain the rectal wall dose level while applying dose escalation to the target of 2−7 Gy. The mean dose to the bulb of penis was also nearly 12 Gy higher on the CT‐delineated plan. Additional to the prostate site, the seminal vesicles are difficult to distinguish from the prostate on CT, and the delineation of the seminal vesicles is also challenged by the nearby ductus deferens and plexular veins.^5^, ^6^, ^7^

MR images reflect the proton density of tissues and they are hence able to better distinguish different soft tissues. Villeirs et al.^(7)^ have shown that MR images are able to reduce the seminal vesicle volume by 10.5%, compared to CT images. The superior soft tissue contrast of MR imaging provides a lower level of interobserver variation^(7)^ and results in more consistent treatment plans with smaller volume margins.^8^, ^9^, ^10^ MR images are typically manually registered with a patient's planning CT to enable prostate contouring. This registration of MR to CT for treatment planning can also introduce geometric errors into treatment planning. For example, using mutual information‐based registration software, Krempien et al.^(11)^ found the CT‐MR registration mean error was 1.8 mm, with a standard deviation of 0.9 mm. In order to reduce the multimodality registration error and simplify treatment workflows, there has been considerable recent interest in the introduction of MRI simulation for prostate radiation therapy where the treatment plan is generated using MRI data alone.^12^, ^13^, ^14^, ^15^ However, there are three significant challenges which need to be addressed before MRI can be used as the sole imaging modality for treatment planning: 1) geometric distortion of the MR image; 2) lack of electron density information for dose calculation; and 3) MR patient positioning system differences from the treatment machine.

MRI distortion results from both system‐ and object‐induced effects, such as magnet inhomogeneity, gradient field nonlinearity, susceptibility, and chemical shift.^(16)^ Previous phantom studies have found that the typical distortion due to machine‐induced effects can be as high as 7−25 mm at 200−260 mm away from the magnet isocenter.^17^, ^18^, ^19^ This is significantly larger than the spatial error tolerance for treatment planning purposes (2 mm^(20)^) and motivates the use of MRI spatial correction before it can be used for planning. Several phantom‐based correction methods have been published^17^, ^18^, ^19^, ^21^ which indicate that the distortion can be corrected to within a submillimeter range.

Electron density is a requirement for radiotherapy treatment planning as it allows the planning software to obtain the relevant attenuation coefficients of the treatment energy. The electron density information can be estimated from the CT Hounsfield units, which provide a representation of tissue linear attenuation coefficients.^(22)^ In image‐guided radiotherapy, patients receive a CT scan (CT simulator) in the treatment position prior to treatment delivery. In contrast to CT, the MR image intensity reflects proton density and the value for the same tissue can vary when different scanning parameters are used, which prevents a unique relationship between electron density and the MR image intensity. Currently there are three main methods to convert MR images into CT‐like images (pseudo‐CT): the first is to manually or automatically identify the main tissue types in the MR image and apply bulk densities to these tissue classes;^(12)^ the second method is to use an anatomical atlas‐based method^(14)^ which uses deformable registration to map one or more MRI atlases to a patient's MR image, and then uses the deformation vectors to map a CT atlas across to the same scan. The Chi‐squared test showed no significant difference between plans generated by the real CT image and the pseudo‐CT image.^(14)^ The third method involves classification, rather than registration, to assign voxels from the MRI with CT values.^(13)^

MR patient positioning is also critical for MR only planning. Currently, diagnostic MR scanners are limited in their ability to provide accurate information for radiotherapy treatment planning. Unlike a CT simulator, the tabletop of a standard MR scanner generally has a different shape from the radiotherapy treatment table. The curved‐couch design of the MR scanner increases the ability to scan larger sized patients, but results in external body deformation in the posterior direction. Other MR tables with flat couch designs feature soft cushioning which could also affect the patient contour. The MR anterior body coil is another source of skin deformation. A surface coil is essential to enhance the signal‐to‐noise ratio (SNR) of the MR image. For typical diagnostic MR scanning, the surface coil consists of a posterior coil located underneath the scanner tabletop and an anterior coil normally strapped around the pelvic region of patient. Compared to the natural shape of the patient body on the treatment table, the MR anterior surface coil can alter the external skin contour and cause anterior and lateral body deformation. An analysis of data from 39 patients receiving radiotherapy for prostate cancer comparing CT with a flat couch and MR with a plastic flat couch insert and strapped anterior coils has been performed. The results showed that the body‐to‐isocenter distance on the CT and MR images can be as different as 15 mm, and the left and right posterior oblique directions had the most external deformation due to the MR positioning.^(12)^

A solution to this problem is to position the coil above the patient body. Kapanen et al.^(15)^ used a homemade plastic coil fixation device to scan prostate patients. Kapanen and colleagues investigated the body contour and rectal wall displacement and proposed that with the same treatment positioning mechanism that MR simulation is suitable to replace CT simulation. MR‐simulation commercial solutions for MR scanners have recently become available from major MR vendors. These generally include a flat radiation therapy style hard couch and coil mounts or bridges to raise the anterior coils above the patient. Laser bridge systems that are MR‐compatible are also available. At our institution, the MR scanner was equipped for MR simulation with dedicated radiotherapy flat couch and coil mounts supplied by CIVCO (CIVCO Medical Solutions, Coralville, IA) and a laser bridge from LAP Lasers (LAP Laser, Luneburg, Germany). These new dedicated MR devices are expensive, and their need and benefit for radiotherapy should be carefully examined. The coil mounts also increase the distance from the patient to the coil and this may have an important impact on the image quality for scanning.

In this study, we have investigated the benefit of MR‐simulation dedicated equipment will have for MR‐based prostate treatment planning using volunteer scans. Our aim is to determine: 1) the external and internal anatomical geometry difference between the conventional MR scan setup and the MR‐simulation setup using the commercial MR‐simulator devices (flat couch and coil mounts); 2) the influence on image quality after applying the commercial MR‐simulator devices; and 3) the dosimetric improvement after unifying the planning MR geometry with the treatment geometry. We are also conducting investigations in other aspects of MRI simulation for prostate radiation therapy, including electron density mapping to MRI scans and distortion in pelvic MRI sequences. However, these are being reported separately.

## II. MATERIALS AND METHODS

### A. MR scanner and sequence

A Magnetom Skyra 3T MR scanner (Siemens AG, Erlingen, Germany) was used for this study. A posterior coil (Spine 32, Siemens) was embedded into the scanner table and a Siemens 18‐channel body matrix surface coil was used on the anterior side of the pelvis. The turbo spin echo (TSE) sequence with a relatively shorter repetition time (TR) 3D image acquisition method was used. A large field of view (LFOV) T2 image that includes the entire pelvis and a small FOV (SFOV) T2 image that includes only the prostate were acquired. Because this study concentrates on clinical effects, the images were postprocessed by the clinical protocols, including using the vendor‐provided Prescan Normalize (Siemens) filter to correct the intensity inhomogeneity across the image, and the geometric distortion of the image was corrected by the vendor‐provided 3D distortion correction algorithm. To compare the quality of images acquired with conventional and radiotherapy dedicated positioning methods, volunteers were scanned with both methods using the same coils and image sequence mentioned above. The parameters of sequences including scan time are listed in Table 1.

**Table 1:** MR scan sequence parameters

	*Matrix*	*Pixel Size (mm)*	*TR (ms)*	*TE (ms)*	α *(°)*	*Time (min)*
LFOV	256×256×128	1.56×1.56×1.56	1200	101	135	5:41
SFOV	320×320×60	0.63×0.63×2	1400	97	135	3:55

### B. Positioning process and scanning

**Figure 1:**
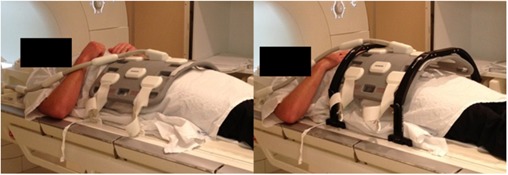
Conventionally the MRI surface coil is strapped onto a patient's pelvis using two pairs of straps (left). The coil induced anterior body deformation can be eliminated by fixing the coil to coil mounts (right).

The CIVCO coil mount consists of two arches to be clipped on the sides of scanner table. The coil can be attached to the arches and suspended above the patient body to a desired height (Fig. 1), so that the external anatomical deformation from the coil strapping in the anterior direction can be eliminated. Each individual arch can be adjusted independently from 30 cm minimum height to 40 cm. This enables the body‐to‐coil distance along the superior–inferior direction to be more consistent for patients with larger waist size, thus improving the overall signal uniformity. The hard flat tabletop is designed to reproduce the patient posterior external geometry at the linear accelerator (linac) treatment table. The Three‐Pin Lok‐Bar accessory, in conjunction with the knee and foot support and the tabletop, is consistent with the 14 cm Varian Exact notched‐style indexing patient positioning system used on the linac treatment table.

Ten healthy volunteers aged below 50 (BMI: 26.9±4.6, mean ±1 SD) have participated in this study. Each volunteer was first positioned on the original scanner table (with embedded table cushion) and the coil was strapped on top of the body. This conventional setup protocol gives a deformed image (I_standard). Then the scanner table was mounted with the CIVCO tabletop and the coil was held above the body using the coil mount to provide an undeformed image (I_MRISim). Depending on the volunteer physical size, the minimum body‐to‐coil distance varied from 1 cm to 5 cm.

### C. Image quality and organ delineation

The signal within the heads of femur (HOF) and bladder on the LFOV image, peripheral zone and central zone of the prostate on the SFOV image were sampled. The mean signals were compared for two setups. The CNR was calculated between different zones of the prostate on the SFOV image. For each of the organs, a linear regression was found based on the ten volunteer scans to obtain an average measure of the organ change.

The signal‐to‐noise ratio (SNR) is defined as the mean intensity of one ROI ($I_{\_\text{mean}}$) to the image background noise ($I_{\_\text{background}}$), which is the standard deviation of a region of air signal. ^23^, ^24^ The contrast‐to‐noise ratio is defined as the difference in intensity between two ROIs ($I_{\_(\text{ROI},1)}-I_{\_(\text{ROI},2)}$) to the image background noise.^23^, ^24^

(1) $$SNR=\frac{I_{mean}}{I_{background}}and\;CNR=\frac{I_{ROI,1}-I_{ROI,2}}{I_{background}}$$

Depending on the individual, a small amount of urine may enter the urethra in some cases. This high intensity on the T2 MR image affects the mean signal calculation of the central zone of the prostate; therefore, any signal that is close to the water signal has been excluded in the prostate mean signal calculation.

The MR images were imported into the Eclipse treatment planning software (version 8.6; Varian Medical Systems, Palo Alto, CA). The prostate was contoured on both SFOV images (I_standard and I_MRISim) by an experienced radiation oncologist. The geometric differences due to the different scanning setups were examined for three aspects: 1) the posterior body deformation; 2) the source‐to‐surface distance (SSD) along the incident beam angles; and 3) the prostate volumes contoured on the SFOV image acquired using two setups.

To measure the posterior body deformation, two horizontal lines were drawn at two levels on the axial image corresponding to the prostate isocenter: one line at the most posterior edge of the body contour to determine the maximum body deformation level, and the second line is drawn across the two lateral sides where the least body deformation occurs.

### D. Dosimetric effect

Three seven‐field 3D conformal plans were created for each volunteer: P_standard, P_MRISim and P_actual. P_standard was generated based on the image acquired with the conventional scan setup (I_standard), while P_MRISim was generated on the image acquired with the CIVCO devices (I_MRISim). P_actual was generated by copying the beam portals and MUs from P_standard to I_MRISim. The rationale of P_actual is to find the dosimetric error caused by planning on a deformed image where the treatment image is actually undeformed. By comparing to P_MRISim, the difference shows the dosimetric improvement of using an undeformed image for planning.

The treatment was planned with 70 Gy prescribed dose to be uniformly delivered in 35 fractions. At each treatment angle, the multileaf collimator was shaped around the target with a uniform margin of 7 mm from the CTV (prostate) to the PTV. The bulk electron density method was used for the dose calculation. Since the aim of this study is to find the dosimetric difference caused by the geometric difference, applying the same bulk density is sufficient. The soft tissue organs were given a water‐equivalent density and the density of head of femurs was set to be $1.19\;g/{\text{cm}}^{3}$ (HU=288).^(12)^ $D_{2},D_{50},D_{95},D_{98},V_{95},V_{98}$, and $V_{100}$ of the prostate were used to compare the plan outcomes.

## III. RESULTS

### A. Image quality and organ delineation agreement

**Figure 2:**
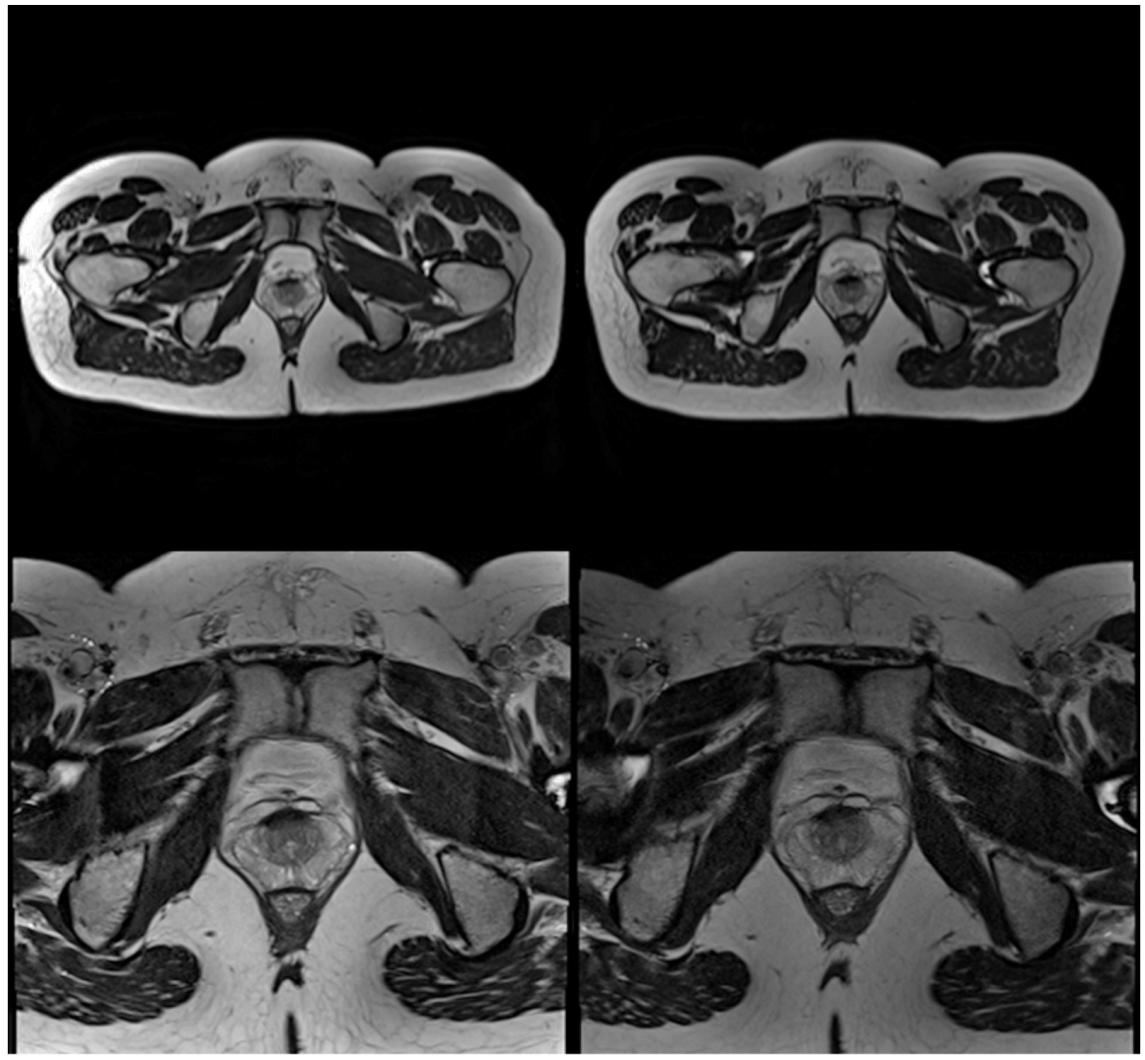
MRI Large field of view (LFOV) (top) and small field of view (SFOV) (bottom) images acquired using conventional scanning protocol, I_standard (left) and radiotherapy‐dedicated protocol, I_MRISim (right). Both images with the same FOV were output with the same window/level setting.

One immediate impact on the image after replacing the conventional setup protocol with the CIVCO devices is the signal loss (Fig. 2). This figure shows the I_standard and I_MRISim scans for one volunteer on an axial slice through the prostate isocenter. The anterior deformation from its natural shape due to the coil strapping can be seen on I_standard. Although the scanner table is flat, the volunteer posterior contour deformation due to the body sink on the soft cushion also can be visualized on the LFOV image.

**Figure 3:**
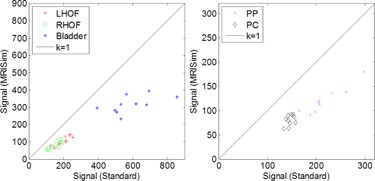
Organ signal comparison on LFOV and SFOV images acquired by the standard setup and MRISim setup. LHOF=left head of femur, RHOF=right head of femur, PP=prostate peripheral zone, PC=prostate central zone.

Figure 3 shows the signal comparison between I_standard and I_MRISim on LFOV and SFOV. The k=1 equality line represents the state when the image qualities from two setups are the same. On average, there was 27%–32.9% mean SNR reduction within the organs of interest (Table 2). The CNR reduction on I_MRISim was 24.4%±17.4%. A radiation oncologist contoured the prostate on the SFOV images acquired by both setups and the mean prostate volume difference for the ten volunteers was $1.05\pm 0.66\;{\text{cm}}^{3}$.

Although the HOFs were located symmetrically along the lateral direction, Fig. 3 shows that the LHOF signal data have drifted away from the RHOF signal data, due to image inhomogeneity. The mean signal ratio of the LHOF to the RHOF was 1.34±0.12 and 1.33±0.08 on the I_standard and I_MRISim images, respectively.

**Table 2:** Mean and standard deviation of the percentage SNR reduction on I_MRISim comparing to I_standard. PP=prostate peripheral zone; PC=prostate central zone

*(%)*	*LHOF*	*RHOF*	*Bladder*	*PP*	*PC*
μ	32.9	32.4	30.4	27.0	27.3
σ	8.7	13.6	17.5	13.1	14.9

### B. Positioning improvement

The SSD (source to surface distance) was determined along the clinical treatment angles on I_standard and I_MRISim images and the SSD difference was determined (Table 3). The positive values at 60° and 300° indicated the anterior coil compression induces longer SSD on the I_standard image. The negative values at 100° and 260° indicate shorter SSD on the I_standard image, which may be due to the anterior coil compression causing lateral body deformation. It should be noted that 0° angle difference is not always as reliable as the other angles, as the penis sometimes appears on the prostate isocenter axial image.

The mean weight of volunteers was 83.4±14.1 kg (ranged from 68 kg to 113 kg). The posterior body deformation due to the body sink on the soft cushion can be visualized on the I_standard image (Fig. 2). The mean maximum posterior body deformation of the ten volunteers was 20.7±2.7 mm.

**Table 3:** Mean and standard deviation of source to surface distance (SSD) difference (mm) along clinical treatment angles between I_standard ‐ I_MRISim

*Angle*	*0*	*60*	*100*	*150*	*210*	*260*	*300*
μ	1.0	2.2	−4.9	0.6	0.7	−4.8	2.8
σ	6.8	3.1	4.5	7.7	8.8	3.9	2.5

### C. Dosimetric effect

**Figure 4:**
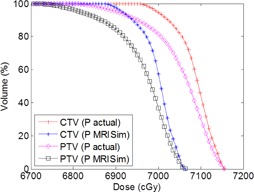
DVH diagram of the one volunteer comparing target dose in P_actual and P_MRISim (reference).

Although there were differences in anatomical geometry on I_standard and I_MRISim, the mean target dose difference between the plans (P_standard and P_MRISim) was only 0.1±0.1 Gy as both plans were normalized to the target isocenter. However, when applying the plan created on the deformed image to the undeformed image, the mean target dose difference between P_MRISim (reference) and P_actual for the ten volunteers increased to 0.6±0.3 Gy. This increased dose difference is the result of the inconsistency between the planning and treatment anatomical geometry. Figure 4 compares target dose from P_MRISim (reference) and P_actual for one volunteer.

## IV. DISCUSSION

As outlined in the Introduction, there are several important aspects to consider for MRI simulation including distortion in the images due to gradient nonuniformities, patient induced susceptibility related distortions, and patient positioning for simulation at MRI. This particular work forms part of a study series investigating different aspects of MRI simulation for prostate therapy and has focused only on the positioning aspect. We have also conducted investigations into the effect of distortions using a custom‐built, pelvic‐shaped distortion phantom and are developing methods to accurately estimate electron densities for MRI scans using atlas based methods. However, these are the subject of separate manuscripts in progress. The investigations of image quality outlined here were performed with volunteer scan comparisons. To gain a more systematic insight into image quality, including scan homogeneity and signal‐noise ratio effects due to MRI coil mounts, we have constructed an anthropomorphic pelvic phantom and varied the scanning geometry and have reported these results separately.^(25)^

A unique feature of this study is that it has investigated the impact of applying a commercial radiotherapy dedicated MR scanner device on image quality. It has also investigated body deformation from both posterior and anterior directions in the standard scanning protocol and determined the dosimetric effect. This study has also supported the necessity of applying a hard tabletop, instead of using a soft one, in order to increase consistency between planning and treatment anatomic geometry.

In this study, the effects of using a coil mount and flat tabletop were investigated in three aspects: image quality, anatomical geometry, and dosimetry. As the volunteers were positioned and scanned within a short period of time, the changes in the image quality should only be due to the increase of the body‐to‐coil distance. As this distance increases by using CIVCO devices, the image SNR and CNR were reduced on both LFOV and SFOV images, compared to the standard scanning protocol. However, the mean prostate volume difference on I_standard and I_MRISim was only $1.05\pm 0.66\;{\text{cm}}^{3}$.

Body deformation can be visually observed on images acquired with the standard scanning setup. Additionally, anterior deformation was found to be in the range of 10−17 mm, depending on the location of coil compression. A posterior deformation ranging from 15.6 mm to 23.4 mm was observed when a soft cushion tabletop was used rather than a hard tabletop. In order to unify the external body contour on the planning image with the contour during the actual treatment, both the MRI coil mount and hard tabletop are essential.

Due to the noticeable external body geometry difference on the images acquired by the standard MRI protocol, the actual dosimetry delivery to the target will be affected if one plans on the externally deformed contour image. A mean difference of 0.6±0.3 Gy was found by comparing P_actual to P_MRISim. To compare the two geometries, bulk density/electron density assignment was used. This is not a gold standard for electron density. The density assigned to bone was based on an earlier study where the density was optimized to match effective depths from CT scans for the planned beam angles.^(12)^ Other studies have found close agreement between bulk density calculations and full density calculations. Eilertsen et al.^(26)^ compared bulk density‐assigned CT scan dose calculation to the full density calculation for ten patients and found an average dose difference of only 0.2%. Our work does not propose the use of bulk density assignment for MR‐based radiotherapy planning due to inaccuracies and the requirement to manually delineate bony anatomy. By applying the same bulk density to both MR images, the dose difference due to HU assignment error can be minimized and the effect of geometric contour differences on the dose can be isolated.

One limitation of this study is the small number sample size because of its tedious scanning setup procedure, but the result will be more reliable if the sample size were increased. One improvement would be to conduct this experiment on actual patients (with their consent).

In terms of SNR reduction, McJury et al.^(27)^ found that the SNR drop in a phantom was 14% when inserting a flat tabletop to a curvature tabletop while keeping the surface coil directly touching the phantom anterior surface. In our study, the anterior body‐to‐coil distance was increased by lifting the coil above the anterior body and the posterior body‐to‐coil distance was also increased by inserting a hard tabletop. The increases in the body‐to‐coil distance may be the cause of the higher SNR drop.

This study has investigated the difference in prostate dosimetry. It would be desirable in future work to also contour the rectum and calculate dose. The commercial coil mounts were able to eliminate the external body deformation by holding the coil away from the patient's body. However, a limitation of the current coil mount design is that its minimum height (30 cm) is relatively large for a smaller size patient. Since this study has shown that the image quality drops as the body‐to‐coil distance increases, image quality will be comparatively poorer for patients with a smaller waist size. Therefore, modification of the coil mount design to reduce the minimum mount height would be useful to enable a wider range of patient body types.

## V. CONCLUSIONS

MR imaging delivers clear soft tissue contrast, allowing radiation oncologists to better delineate organs of interest. However, patient anatomical geometry from a conventional MR scanning protocol has been shown to be different to the natural geometry acquired using a hard tabletop. Inserting soft cushions is not recommended as it will induce posterior deformation. Due to the increase in the body‐to‐coil distance when lifting the coil away from the body and inserting a hard tabletop, a noticeable reduction in image quality was shown on MR‐simulator images. Despite this reduction in image quality, coil mounts and hard tabletops are necessary to unify the planning and treatment anatomic geometry. The actual prostate dose delivery was lower than the planning dose if a standard MR acquisition protocol was used, due to the reduced external body contour deformation.

## ACKNOWLEDGMENTS

This work was supported by Cancer Council New South Wales Research Grant RG11‐05. We would like to thank the ten volunteers who gave their time for the MRI scanning.
